# Metal Nanoparticles as Novel Antifungal Agents for Sustainable Agriculture: Current Advances and Future Directions

**DOI:** 10.3390/jof7121033

**Published:** 2021-12-01

**Authors:** Aida R. Cruz-Luna, Heriberto Cruz-Martínez, Alfonso Vásquez-López, Dora I. Medina

**Affiliations:** 1 Instituto Politécnico Nacional, CIIDIR-OAXACA, Hornos Núm 1003, Col. Noche Buena, Santa Cruz Xoxocotlán 71230, Mexico; luna_060877@hotmail.com; 2 Tecnológico Nacional de México, Instituto Tecnológico del Valle de Etla, Abasolo S/N, Barrio del Agua Buena, Santiago Suchilquitongo 68230, Mexico; heri1234@hotmail.com; 3 Tecnologico de Monterrey, School of Engineering and Sciences, Atizapan de Zaragoza 52926, Mexico

**Keywords:** metallic nanoparticles, agriculture, crop protection, antifungal activities, fungi

## Abstract

The use of metal nanoparticles is considered a good alternative to control phytopathogenic fungi in agriculture. To date, numerous metal nanoparticles (e.g., Ag, Cu, Se, Ni, Mg, and Fe) have been synthesized and used as potential antifungal agents. Therefore, this proposal presents a critical and detailed review of the use of these nanoparticles to control phytopathogenic fungi. Ag nanoparticles have been the most investigated nanoparticles due to their good antifungal activities, followed by Cu nanoparticles. It was also found that other metal nanoparticles have been investigated as antifungal agents, such as Se, Ni, Mg, Pd, and Fe, showing prominent results. Different synthesis methods have been used to produce these nanoparticles with different shapes and sizes, which have shown outstanding antifungal activities. This review shows the success of the use of metal nanoparticles to control phytopathogenic fungi in agriculture.

## 1. Introduction

Since the beginning of agriculture, the biggest challenge has been pests and diseases produced by insects, bacteria, fungi, and other pathogens present in the environment [1,2,3]. This leads to large losses of crops, which are reflected in production with low profits, that is to say, earnings are directly affected [4,5]. Among the different pathogens, phytopathogenic fungi cause various diseases in agriculture [6]. Fungi have the versatility of adaptation to any medium and are capable of colonizing different substrates or media in extreme or precarious environmental conditions. They can affect different stages of the crop, from sowing to growth and production to postharvest [7,8].

Today, phytopathogenic fungi have mostly been controlled with chemical products, which are cheap and easy to obtain on the market [9,10]. However, due to their indiscriminate use, they have created several problems such as environmental pollution, diseases in humans and animals, and ecological imbalances [11,12]. In addition, the usage of chemical agents has resulted in fungi developing more resistance, becoming stronger against chemical products [13,14].

Currently, friendly and efficient alternatives for the environment are being used to control phytopathogen fungi, such as biological control [15,16], plant extracts [17], and essential oils [18,19,20]. Such alternatives have been beneficial and are therefore considered as a good choice. However, these alternatives have some challenges, such as the effect of delays, high acquisition costs, and constant applications that make them vulnerable [21,22].

Otherwise, another recently explored and applied route in agriculture is the use of nanomaterials, which have been successfully applied in other fields such as energy, medicine, and electronics [23,24,25,26,27]. Nanomaterials have become very important because their physicochemical properties are very different compared to bulk materials [28,29,30]. Furthermore, the shape, size, and composition of nanomaterials determine their physicochemical properties [28,29,30]. These peculiarities have made nanomaterials applicable in different areas. Specifically, in the field of agriculture, there are several nanomaterial applications, such as in the production, processing, storage, packaging, and transportation of agricultural products [31,32]. In production, nanomaterials offer ecological, efficient, and modern alternatives that can be very useful for the management of phytopathogenic diseases that can be used as bio-manufacturing agents, due to their easy handling and production [33,34].

Different nanomaterials have shown excellent antifungal activities; therefore, they are considered a good alternative to control phytopathogenic fungi [35,36,37,38]. Specifically, metal nanoparticles have been widely studied; consequently, they have been tested and led to significant results due to their excellent antifungal properties [39]. So far, numerous metal nanoparticles have been synthesized and used to control phytopathogenic fungi [40,41,42,43,44,45,46,47]. However, there is a current lack of critical and detailed reviews of current progress in the use of metal nanoparticles to control phytopathogenic fungi, as the currently available reviews only partially analyse the use of metal-based nanoparticles for controlling these pathogens [48,49].

Therefore, this review presents a comprehensive and detailed analysis of the current progress on the application of metal nanoparticles for controlling phytopathogenic fungi in agriculture. In the first instance, the possible mechanisms of action of nanoparticles on phytopathogenic fungi are reviewed. Afterwards, the progress on the use of metal nanoparticles as potential antifungal agents is reviewed in detail. Finally, conclusions and future directions are presented.

## 2. Mechanisms Involved in Antifungal Activity of Nanoparticles

The use of nanoparticles is a novel route to control phytopathogenic fungi in agriculture because they have shown high antifungal activity across a wide diversity of phytopathogenic fungi [50,51]. Several factors have an influence over their antifungal activity, such as the size distribution, shape, composition, crystallinity, agglomeration, and surface chemistry of the nanoparticles [52,53]. For example, small nanoparticles favor the surface area-to-volume ratio, which could promote their antifungal activity [54]. It is well-known that these mentioned factors can be modified and controlled through synthesis routes [55,56]. It has also been documented that the synthesis route can play an important role in the antifungal activity, as sometimes metal precursors or surfactants are not easy to remove from the nanoparticles. Therefore, the residues from the synthesis can modify the surface chemistry of the nanoparticles and consequently influence their antifungal activity [57]. Finally, another important factor is the species of phytopathogenic fungi, since each specie has a different morphological structure.

**Figure 1:**
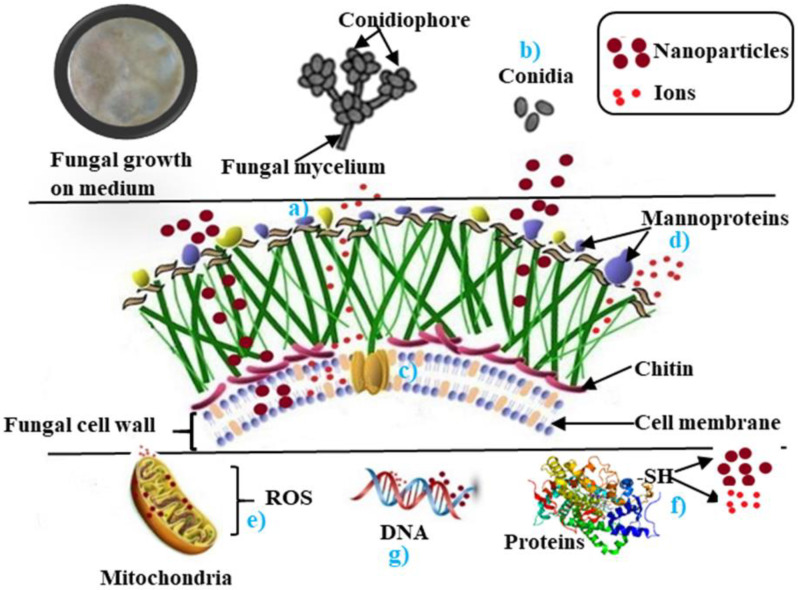
This is an illustration of the possible mechanisms of action of metal nanoparticles on phytopathogenic fungi. These are as follows: (**a**) ions are released by nanoparticles and bind to certain protein groups, which affect the function of essential membrane proteins and interfere with cell permeability. (**b**) The nanoparticles inhibit the germination of the conidia and suppress their development. (**c**) Nanoparticles and released ions disrupt electron transport, protein oxidation, and alter membrane potential. (**d**) They also interfere with protein oxidative electron transport. (**e**) They affect the potential of the mitochondrial membrane by increasing the levels of transcription of genes in response to oxidative stress (ROS). (**f**) ROS induces the generation of reactive oxygen species, triggering oxidation reactions catalyzed by the different metallic nanoparticles, causing severe damage to proteins, membranes, and deoxyribonucleic acid (DNA), and interfering with nutrient absorption. (**g**) The ions of the nanoparticles have a genotoxic effect that destroys DNA, therefore causing cell death [58,59,60,61,62].

As mentioned before, several factors influence the antifungal activity of the nanoparticles. Therefore, it is necessary to know the interaction and action mechanism between the metal nanoparticles and the phytopathogenic fungi. At present, various possible antifungal action mechanisms of these nanoparticles have been proposed (see Figure 1).

## 3. Antifungal Properties of Metal Nanoparticles

Metal nanoparticles have been successfully applied to control different pathogens [63,64,65]. In this same direction, there are numerous studies on the use of metal nanoparticles to control phytopathogenic fungi in agriculture. Up to now, different nanoparticles have been used to control phytopathogenic fungi. For instance, Ag, Cu, Fe, Zn, Se, Ni, and Pd have shown outstanding antifungal properties. Therefore, a critical and detailed analysis of current advances in the use of metal nanoparticles on phytopathogenic fungi is presented.

### 3.1. Ag Nanoparticles

**Figure 2:**
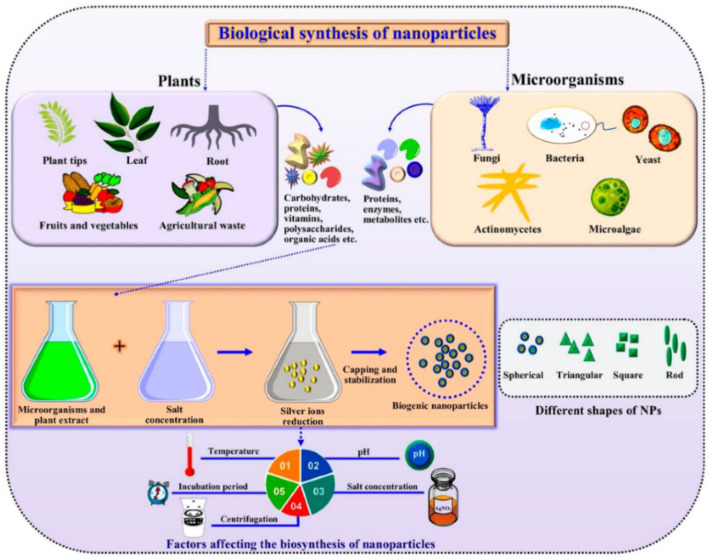
A generalized representation of the green synthesis of metallic nanoparticles [75].

Ag nanoparticles have been extensively investigated in different scientific fields due to their antioxidant, antimicrobial, and anticancer properties as well as their characteristics of biocompatibility, easy production, relatively low cost, and non-toxicity, among others [66,67,68,69,70,71,72]. Due to these properties and their effective antifungal activities, Ag nanoparticles have also been the most investigated nanoparticles to control phytopathogenic fungi [73,74]. The main synthesis methods used to produce Ag nanoparticles to inhibit the growth of phytopathogenic fungi are the chemical and biological routes because they are easy to acquire and handle. In Figure 2, a generalized representation of the green or biological synthesis of metallic nanoparticles is illustrated. It can be observed that several factors can influence the synthesis of nanoparticles.

For biological systems, many extracts of plants and fungi have been used in the synthesis of Ag nanoparticles [33,76,77,78,79,80,81,82,83,84,85,86,87,88,89,90,91,92,93,94,95,96,97,98,99,100,101,102,103,104,105,106,107,108,109,110,111,112,113,114,115,116,117,118,119,120,121]. In Table 1, the different extracts of plants and fungi that have been used to produce Ag nanoparticles are reported. In the case of the chemical route, several methods have been used to synthesize Ag nanoparticles, such as chemical reduction, sol-gel, and microemulsion [122,123,124,125,126,127,128,129,130]. To a lesser extent, physical methods have been used, such as high-voltage arc discharge and the irradiation method [131,132,133]. These different methods have made it possible to synthesize Ag nanoparticles with outstanding antifungal properties. Moreover, the biological syntheses present an additional benefit because they are environmentally friendly. Finally, it is interesting to note that several commercial Ag nanoparticles have been evaluated to inhibit the growth of phytopathogenic fungi, and have shown outstanding antifungal properties.

**Figure 3:**
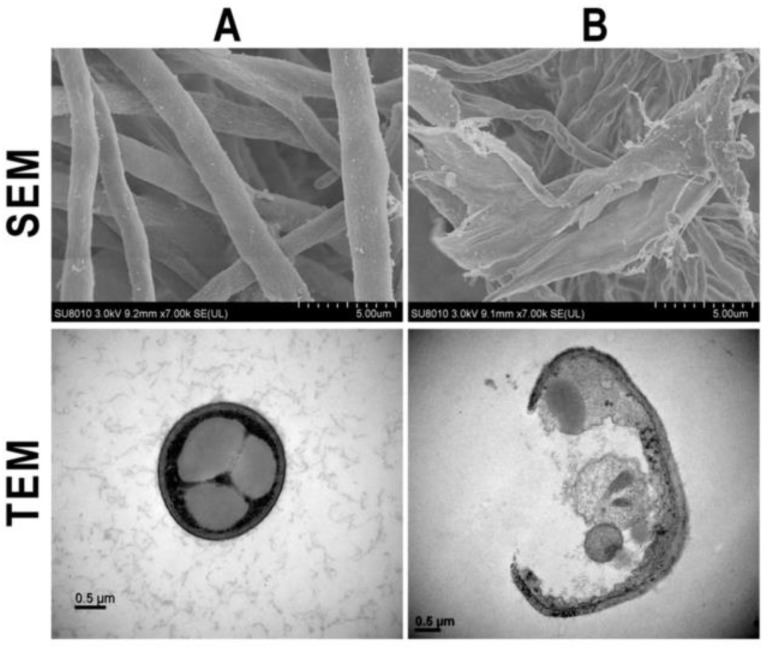
Microscopic images of SEM and TEM of *F. graminearum* in the absence (**A**) and presence (**B**) of the synthesized silver nanoparticle [116].

As aforementioned, the characteristics of Ag nanoparticles such as shape, structure, and size play an important role in antifungal activity. According to Table 1, so far, most Ag nanoparticles synthesized by the different methods have been spherical, which may be because this kind of shape is easier to synthesize. In terms of size, they are polydisperse, which does not allow analysis in detail of the effect of the size of the nanoparticles on their antifungal activity. However, it is revealed that the smaller nanoparticles, between 10 and 30 nm, have greater antifungal effectiveness [76,77,90,94,99,104,108,126,133,148]. This is because the smaller nanoparticles penetrate or destroy the pathogen’s cell membrane more quickly and thus unite the fungal hyphae and mycelium and deactivate these pathogens [99,108]. Ag nanoparticles ranging between 40 and 70 nm also show an inhibitory effect, destroying mycelium and spores and provoking the rupture of the membrane significantly [78,92,95,118,122,131]. Nevertheless, while the larger size has a good antifungal capacity, their penetration into the pathogen’s membrane is slower, causing damage to mycelium and spores or the inhibition of fungal growth [110,121,129,132]. In Figure 3, severely damaged cell walls and hyphae with abnormal structures are shown in the presence of biosynthesized Ag NPs.

On the other hand, it has been reported that the concentration of nanoparticles can play an important role in antifungal activity [130]. Therefore, different concentrations of Ag nanoparticles have been evaluated. Several studies have shown that the concentration of Ag nanoparticles has an important role in antifungal activity [83,113,129,130,137,146]. Interestingly, low concentrations showed effectiveness in the suppression of fungi. For example, Ag nanoparticles synthesized with *M. charantia* and *P. guajava* extracts showed good antifungal capacity in concentrations of 20 ppm, inhibiting the growth of mycelium in fungi such as *A. niger*, *A. flavus*, and *F. oxysporum* [76]. A similar case occurred with Ag nanoparticles synthesized with *T. viride* extracts, which completely inhibited the growth of *A. solani* at low concentrations of 25 ppm [103]. In addition, excellent results were found in medium concentrations. For example, Ag nanoparticles synthesized with green and black tea were evaluated in four concentrations (i.e., 10, 25, 50, and 100 ppm) against *A. flavus* and *A. parasiticus*. The best results were obtained with doses of 100 ppm. Ag nanoparticles entered into the cell membrane, seriously affecting the respiratory chain, resulting in cell death [90]. A peculiarity was observed at very high concentrations of Ag nanoparticles (e.g., 500, 1000, 5000, and 10,000 ppm): with the increasing dose, the antifungal capacity presented a saturation of the Ag nanoparticles. According to the literature, this caused damage to the mycelium, such as oxidation, but not the complete inhibition of fungal pathogens [80,99,107]. Interestingly, some studies compare the antifungal activities of Ag nanoparticles with respect to chemical fungicides [109,144]. Ag nanoparticles showed similar results to chemical fungicides [109,144]. Therefore, the utilization of nanoparticles is a viable alternative to the use of chemical fungicides.

### 3.2. Cu Nanoparticles

The first study of Cu nanoparticles against fungi was reported by Giannousi et al. [149]. Since then, Cu nanoparticles have been considered a viable option for the treatment of fungal diseases [150,151]. Furthermore, Cu has several advantages: for instance, it is cheap, it is highly available, and its production in terms of nanoparticles is economical. Therefore, there are several studies on the use of Cu nanoparticles on phytopathogenic fungi [42,79,90,92,152,153,154,155,156,157,158,159,160,161,162,163,164,165]. The main synthesis methods to obtain Cu nanoparticles for the control of this pathogen are mentioned in Table 2. The chemical synthesis methods include chemical reduction and hydrothermal [158,159,160,161,162,163,164], whereas biological synthesis with different extracts of plants is widely used for its naturalness and its zero toxicity concerning the environment [42,90,92,154,155,156]. Finally, commercial nanoparticles, which are effective and easily acquired, have also been evaluated for the inhibition of phytopathogenic fungi [139,140,142,145,165].

**Figure 4:**
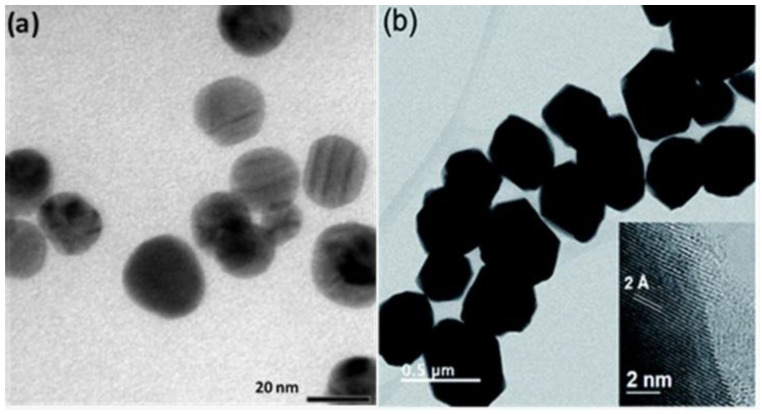
Cu nanoparticles synthesized with different shapes and sizes: (**a**) spherical shapes [158] and (**b**) faceted shapes [152].

The studies carried out on Cu nanoparticles produced by the different synthesis methods have shown excellent antifungal activity in different species of phytopathogenic fungi. However, as in the case of Ag nanoparticles, there is a great diversity of sizes, which makes it difficult to analyze the size effect of Cu nanoparticles on antifungal activity (see Figure 4). In general, small nanoparticles range from 10 to 30 nm and penetrate the cell membrane more easily, causing a rupture and the leakage of cell contents [139,142,145,154,165]. Something similar occurs in medium-sized Cu nanoparticles (40 to 70 nm); however, by increasing their size, their fluidity in the membrane makes the growth and development of colonies of the pathogen impossible [90,92,158]. Finally, the large Cu nanoparticles (80 to >100 nm) inhibit the growth of mycelium and spores, thus demonstrating their antifungal capacity [152,153,161].

Regarding the shape, the synthesized Cu nanoparticles are mainly spherical (see Figure 4a). That kind of shape has shown outstanding antifungal activities. According to several authors, spherical nanoparticles have the highest possibility of penetrating the membrane (and thus accessing the enzymes to initiate the cellular inhibition) faster [145,162]. Other shapes were also found, such as faceted ones with sizes in the range of 200–500 nm, which showed high effectiveness against *F. solani*, *Neofusicoccum* sp., and *F. oxysporum* (see Figure 4b) [152]. Another shape is the truncated octahedron structure (14 to 37 nm), which has been effective against *F. oxysporum* and caused its inhibition [164].

Another determining factor in inhibiting the growth of phytopathogenic fungi is the concentration of the Cu nanoparticles. To date, different concentrations (e.g., low, medium, and high) have been evaluated on phytopathogenic fungi. For example, low concentrations of Cu nanoparticles were evaluated against *F. oxysporum* at 0.1, 0.25, and 0.5 ppm. While the lowest concentration (0.1 ppm) promoted hard oxidative stress in the mycelium, the highest concentration (0.5 ppm) showed an antifungal capacity against *F. oxysporum* [164].

In addition, they have antifungal activities at medium concentrations (e.g., 5, 10, and 20 ppm). Cu nanoparticles demonstrated significant antifungal activity against *F. oxysporum* and *P. capsici*, which were inhibited by increasing the incubation time of the different concentrations. On the third day after their application, the inhibition increased slightly from 49% for 5 ppm to 63% for 20 ppm [157].

To cite another example, doses of 5, 15, 25, and 35 ppm were used against *R. solani*, *F. oxysporum*, *F. redolens*, *P. cactorum*, *F. hepática*, *G. frondosa*, *M. giganteus*, and *S. crispa*, demonstrating the antifungal capacity of Cu nanoparticles at a concentration of 35 ppm. In such a case, there was neither the growth of mycelium, nor the development of the pathogens studied [140]. Finally, for the highest concentrations of Cu nanoparticles, three different doses (300, 380, and 450 ppm) were evaluated. They were applied against *Fusarium* sp., demonstrating excellent antifungal capacity at the highest dose of 450 ppm [158]. Another study was carried out at four different high doses (i.e., 50, 100, 500, and 1000 ppm) against *B. cinerea*, *A. alternata*, *M. fructicola*, *C. gloeosporioides*, *F. solani*, *F. oxysporum*, and *V. dahlia*. In this study, Cu nanoparticles showed toxic activity at all concentrations and at the highest concentration of 1000 ppm they inhibited all phytopathogens [139]. In general, the Cu nanoparticles show antifungal capacity, affecting the phytopathogen intracellularly and extracellularly. Therefore, Cu nanoparticles are an excellent option for the control and management of different diseases of agronomic importance.

### 3.3. Other Metal Nanoparticles

As previously discussed, Ag and Cu nanoparticles are the most studied for the control of the growth of phytopathogenic fungi. However, other metal nanoparticles have been investigated as antifungal agents, such as Se [103,129,166], Ni [47,92], Mg [92], Pd [167], and Fe [90], which have shown promising results. Recently, Se nanoparticles were evaluated in vivo against *S. graminicola* in doses of 0 to 1000 ppm. To synthesize these nanoparticles, six strains of Trichoderma spp. (*T. asperellum*, *T. harzianum*, *T. atroviride*, *T. virens*, *T. longibrachiatum*, and *T. brevicompactum*) in the form of culture filtrate, cell lysate, and crude cell wall were used. The best result was found with *T. asperellum* in culture filtrate, demonstrating the antifungal capacity of Se nanoparticles [166]. In another report, Se nanoparticles were synthesized by the biological method using *T. viride* and they were evaluated at different concentrations (50, 100, 200, 300, 400, 500, 600, 700, and 800 ppm) against *A. solani* using the in vitro method. It was demonstrated that Se nanoparticles suppressed the growth of the fungus at 800 ppm [103]. Lastly, chemically synthesized Se nanoparticles were evaluated against *M. phaseolina, S. sclerotiorum*, and *D. longicolla* at different concentrations of 0.1, 0.5, 1, 5, 10, 50, and 100 ppm. The nanoparticles of Se inhibited *D. longicolla* from 10 ppm and up, and from 50 and 100 ppm for *M. phaseolina*. However, for *S. sclerotiorum*, the different concentrations of Se nanoparticles did not show any inhibition, allowing the growth and development of the pathogen [129].

Another metal that has been investigated for the control of phytopathogenic fungi is Ni. However, as in the case of Se nanoparticles, there are few studies available on the use of Ni nanoparticles aganist phytopathogenic fungi. In the first instance, commercial Ni nanoparticles were evaluated using in vitro and in vivo methods against two species of *F. oxysporum* at concentrations of 50 and 100 ppm. At a concentration of 100 ppm, the Ni nanoparticles significantly inhibited mycelial reproduction and the sporulation activities of the fungal pathogens under in vitro conditions. Meanwhile, under in vivo conditions, Ni nanoparticles at a concentration of 50 ppm reduced the severity of the disease by 58.4% and 57.0% in the cases of lettuce and tomato crops [47].

Finally, other nanoparticles investigated for the control of phytopathogenic fungi are Fe nanoparticles, highlighting the application of Fe nanoparticles synthesized by an ecological method using extracts of green and black tea leaves. Various concentrations (10, 25, 50, and 100 ppm) were evaluated against the fungi *A. flavus* and *A. parasiticus* in vitro. The results demonstrated a 43.5% inhibition with green tea extract and a 51.6% inhibition with black tea with doses of 100 ppm [90].

## 4. Conclusions and Future Directions

In this review, a critical and detailed analysis of the current progress on the application of metal-based nanoparticles for controlling phytopathogenic fungi in agriculture was presented. Based on this review, the following conclusions and future directions are proposed.

The progress achieved in the use of metal nanoparticles for the control of phytopathogenic fungi is outstanding since the studies developed so far clearly show that these nanoparticles can be an excellent alternative to chemical fungicides for the control of phytopathogenic fungi in agriculture.

Among the metallic nanoparticles, Ag nanoparticles have been the most studied as antifungal agents, followed by Cu nanoparticles. These nanoparticles have shown promising activity aganist different species of phytopathogenic fungi. Different synthesis methods have made it possible to produce nanoparticles with different shapes and sizes. However, the nanoparticles have been mainly spherical and polydisperse in size. Therefore, we consider it necessary to synthesize and evaluate nanoparticles of different shapes and size (e.g., octahedrons, icosahedrons, and faceted ones) and homogeneous in, since it is well known that these factors influence on antifungal activity.

For the rest of the metallic nanoparticles, such as Ni, Se, Mg, Pd, and Fe, there is little research. Therefore, it can be inferred that their antifungal properties are not well known, although the synthesis methods that have been tested for them have given good results. Hence, it is important to continue researching these metallic nanoparticles since there is a vast number of opportunities for researchers in this field.

Nowadays, the nanoparticles evaluated as antifungal agents have been mainly monometallic. Therefore, we consider it important to synthesize and evaluate bimetallic or trimetallic nanoparticles for the control of phytopathogenic fungi, since it has been documented that these nanoparticles have very different properties than monometallic nanoparticles.

According to this review, most of the studies were evaluated in vitro. However, it is important to apply the in vivo method to know the behavior of phytopathogens in the field. Applying the nanoparticles directly to the pathogens is preferable since the environments within the laboratory are different from those in the field. The lack of in vivo studies create a significant opportunity for the application of metal nanoparticles in the field of agriculture.

## Figures and Tables

**Table 1:** Characteristics and antifungal evaluations of Ag nanoparticles.

Nanoparticle Properties			Antifungal Properties		Ref.
Synthesis Method	Size (nm)	Shape	Specie of Fungi	Evaluation Method	
Biological synthesis (*M. charantia* and *P. guajava*)	17 and 25.7	Spherical	*A. niger*, *A. flavus*, and *F. oxysporum*	In vitro	[76]
Biological synthesis (*M. azedarach*)	23	Spherical	*V. dahliae*	In vitro and in vivo	[77]
Biological synthesis (*A. indica*)	10–50	Spherical	*A. alternata*, *S. sclerotiorum*, *M. phaseolina*, *R. solani*, *B. cinerea*, and *C. lunata*	In vitro	[78]
Biological synthesis (*A. officinalis*, *T. vulgaris*, *M. pulegium*)	50	Spherical	*A. flavus* and *P. chrysogenum*	In vitro	[79]
Biological synthesis (*S. hortensis*)	-	-	*F. oxysporum*	In vitro	[80]
Biological synthesis (*O. fragrans*)	20	Spherical	*B. maydis*	In vitro	[81]
Biological synthesis (*P. glabra*)	29	Spherical	*R. nigricans*	In vitro	[82]
Biological synthesis (*W. somnifera*)	10–21	Spherical	*F. solani*	In vitro and in vivo	[83]
Biological synthesis (*P. vulgaris*)	12–16	Spherical	*Colletotrichum* sp., *F. oxysporum*, *F. acuminatum*, *F. tricinctum*, *F. graminearum*, *F. incarnatum*, *R. solani*, *S. sclerotiorum*, and *A. alternata.*	In vitro	[84]
Biological synthesis (*V. amygdalina*)	-	-	*F. oxysporum*, *F. solani*, and *C. canescent*	In vitro	[85]
Biological synthesis (*Z. officinale*)	75.3	Spherical	*A. alternata* and *C. lunata*	In vitro	[86]
Biological synthesis (*C. sinensis*)	-	-	*Irenopsis* spp., *Diaporthe* spp., and *Sphaerosporium* spp.	In vitro	[87]
Biological synthesis (*A. absinthium*)	-	-	*P. parasitica*, *P. infestans*, *P. palmivora*, *P. cinnamomi*, *P. tropicalis*, *P. capsici*, and *P. katsurae*	In vitro and in vivo	[88]
Biological synthesis (*M. parviflora*)	50.6	Spherical	*H. rostratum*, *F. solani*, *F. oxysporum*, and *A. alternata*	In vitro	[89]
Biological synthesis (Green and black teas)	10–20	Spherical	*A. flavus* and *A. parasiticus*	In vitro	[90]
Biological synthesis (*P. shell*)	10–50	Spherical and oval	*P. infestans* and *P. capsici*	In vitro	[91]
Biological synthesis (Ajwain and neem)	68	-	*C. musae*	In vitro and in vivo	[92]
Biological synthesis (*T. patula*)	15–30	Spherical	*C. chlorophyti*	In vitro and in vivo	[93]
Biological synthesis (*A. retroflexus*)	10–32	Spherical	*M. phaseolina*, *A. alternata*, and *F. oxysporum*	In vitro	[94]
Biological synthesis (*T. majus*)	35–55	Spherical	*A. niger*, *P. notatum*, *T. viridiae*, and *Mucor* sp.	In vitro	[95]
Biological synthesis (*T. foenum-graecum*)	20–25	Spherical	*A*. *alternata*	In vitro	[96]
Biological synthesis (Rice leaf)	3.7–29.3	Spherical	*R. solani*	In vitro	[97]
Biological synthesis (*P. urinaria*, *P. zeylanica*, and *S. dulcis*)	4–53	Various morphologies	*A. niger*, *A. flavus*, and *F. oxysporum*	In vitro	[98]
Biological synthesis (*C. globosum*)	11 and 14	Spherical	*F. oxysporum*	In vivo and in vitro	[99]
Biological synthesis (*T. longibrachiatum*)	10	Spherical	*F. verticillioides*, *F. moniliforme*, *P. brevicompactum*, *H. oryzae*, and *P. grisea*	In vitro	[100]
Biological synthesis (*A. terreus*)	5–30	Spherical	*A. flavus*	In vitro	[101]
Biological synthesis (*F. oxysporum*)	10–30	Spherical	*P. aphanidermatum*	In vitro and in vivo	[102]
Biological synthesis (*T. viride*)	12.7	Spherical	*A. solani*	In vitro	[103]
Biological synthesis (*F. solani*)	5–30	Spherical	*F. oxysporum*, *F. moniliform*, *F. solani*, *F. verticillioides*, *F. semitectum*, *A. flavus*, *A. terreus*, *A. niger*, *A. ficuum*, *P. citrinum*, *P. islandicum*, *P. chrysogenum*, *R. stolonifer*, *Phoma*, *A. alternata*, and *A. chlamydospora*	In vitro	[104]
Biological synthesis (*B. subtilis*)	16–20	Spherical	*A. alternate*, *A. niger*, *A. nidulans, C. herbarum*, *F. moniliforme*, *Fusarium* spp., *F. oxysporum*, and *T. harzianum.*	In vitro	[105]
Biological synthesis (*B. pseudomycoides*)	25–43	Spherical	*A. flavus*, *A. niger*, *A. tereus*, *P. notatum*, *R. olina*, *F. solani*, *F. oxysporum*, *T. viride*, *V. dahlia*, and *P. spinosum*	In vitro	[106]
Biological synthesis (*T. harzianum*)			*F. moniliforme*	In vitro	[107]
Biological synthesis (*Alternaria* sp.)	3–10	Spherical	*Alternaria* sp., *F. oxysporum*, *F. moniliforme*, and *F. tricinctum*.	In vitro	[108]
Biological synthesis (*Bacillus* sp.)	22.33–41.95	Spherical	*C. falcatum*	In vitro	[109]
Biological synthesis (*C. laurentii* and *R. glutinis*)	15–400	Spherical	*B. cinerea*, *P. expansum*, *A. niger*, *Alternaria* sp., and *Rhizopus* sp.	In vitro	[110]
Biological synthesis (*A. foetidus*)	20–40	Spherical	*A. niger*, *A. foetidus*, *A. flavus*, *F. oxysporum*, *A. oryzae*, and *A. parasiticus*	In vitro	[111]
Biological synthesis (*P. verrucosum*)	10–12	Spherical	*F. chlamydosporum* and *A. flavus*	In vitro	[112]
Biological synthesis (*N. oryzae*)	3–13	Spherical	*F.sambucinum*, *F.semitectum*, *F.sporotrichioides*, *F.anthophilium*, *F.oxysporum*, *F.moniliforme*, *F.fusarioids*, and *F.solani*	In vitro	[113]
Biological synthesis (*T. longibrachiatum*)	1–20	Spherical	*F. oxysporium*	In vitro	[114]
Biological synthesis (*A. versicolor*)	5–30	Spherical	*S. sclerotiorum* and *B. cinerea*	In vitro	[115]
Biological synthesis (*P. poae*)	19.8–44.9	Spherical	*F. graminearum*	In vitro	[116]
Biological synthesis (*Alternaria* spp.)	5–10	Spherical	*F. oxysporum*, *F. maniliforme, F. tricinctum*, and *Alternaria* sp.	In vitro	[117]
Biological synthesis (*I. hispidus*)	69.24	-	*Pythium* sp., *A. niger*, and *A. flavus*	In vitro	[118]
Biological synthesis (*S. griseoplanus*)	19.5–20.9	Spherical	*M. phaseolina*	In vitro	[119]
Biological synthesis (Sodium alginate)	6 and 40	Spherical	*C. gloeosporioides*	In vitro	[120]
Biological synthesis (*F. oxysporum)*	93 ± 11	Spherical	*A. flavus*, *A. nomius*, *A. parasiticus*, *A.ochraceus*, and *A. melleus*	In vitro	[121]
Biological synthesis (Glucose)	5–24	Spherical	*C. gloesporioides*	In vitro	[33]
Chemical synthesis	40–60	Spherical	*R. solani*	In vitro	[122]
Chemical synthesis	21 ± 2	Spherical	*C. gloeosporioides*	In vitro	[123]
Chemical synthesis	52	Spherical	*Phomopsis* sp.	In vitro	[124]
Chemical synthesis	30	Spherical	*F. graminearum*, *F. culmorum*, *F. sporotrichioides*, *F. langsethiae*, *F. poae*, *F. oxysporum*, *F. proliferatum*, and *F. verticillioides*	In vitro	[125]
Chemical synthesis	19–24	Spherical	*C. gloeosporioides*	In vitro	[126]
Chemical synthesis	25–32	-	*B. sorokiniana* and *A. brassicicola*	In vitro	[127]
Chemical synthesis	20	Spherical	*A. parasiticus*	In vitro	[128]
Chemical synthesis	100	Spherical	*M. phaseolina*, *S. sclerotiorum*, and *D. longicolla.*	In vitro	[129]
Chemical synthesis	-	-	*A. citri*	In vitro	[130]
Chemical synthesis	47	Spherical	*C. gloeosporioides*	In vitro	[134]
Commercial	7–25	-	*A. alternata*, *A. brassicicola*, *A. solani*, *B. cinerea*, *C. cucumerinum*, *C. cassiicola*, *C. destructans*, *D. bryoniae*, *F. oxysporum* f. sp. *cucumerinum*, *F. oxysporum* f. sp. *lycopersici*, *F. oxysporum*, *F. solani*, *Fusarium* sp., *G. cingulata, M. cannonballus*, *P. aphanidermatum P. spinosum*, and *S. lycopersici*	In vitro	[135]
Commercial	20–30	-	*B. sorokiniana* and *M. grisea*	In vitro and in vivo	[136]
Commercial	-	-	*R. solani*, *M. phaseolina*, *S. sclerotiorum*, *T. harzianum*, and *P. aphanidermatum*	In vitro and in vivo	[137]
Commercial	20	-	*S. homoeocarpa*	In vitro	[138]
Commercial	<100	-	*B. cinerea*, *A. alternata*, *M. fructicola*, *C. gloeosporioides*, *F. solani*, *F. oxysporum* f. sp. *Radicis* *Lycopersici*, and *V. dahliae*	In vitro and in vivo	[139]
Commercial	-	-	*R. solani*, *F. oxysporum*, *F. redolens*, *P. cactorum*, *F. hepática*, *G. frondosa*, *M. giganteus* and *S. crispa*	In vitro	[140]
Commercial	40–50	Spherical	*A. flavus*	In vitro	[141]
Commercial	20–30	-	*S. carvi*	In vitro and in vivo	[142]
Commercial	<100	-	*M. fructicola*	In vitro and in vivo	[143]
Commercial	4–8	-	*Colletotrichum*	In vitro and in vivo	[144]
Commercial	38	Spherical	*A. alternata* and *B. cinerea*	In vitro	[145]
Commercial	7–25	-	*S. cepivorum*	In vitro	[146]
Commercial	-	-	*B. cinerea*	In vitro and in vivo	[147]
Commercial	5–10	-	*R. solani*	In vitro and in vivo	[148]
Physical synthesis	5–65	Spherical	*F. culmorum*	In vitro	[131]
Physical synthesis	15–100	Spherical	*F. culmorum*	In vitro	[132]
Physical synthesis	5–15	Spherical	*P. capcisi*	In vitro and in vivo	[133]

**Table 2:** Characteristics and antifungal evaluations of Cu nanoparticles.

Nanoparticle Properties			Antifungal Properties		Ref.
Synthesis Method	Size (nm)	Shape	Specie of Fungi	Evaluation Method	
Biological synthesis (*Persea americana*)	42–90	Spherical	*A. flavus*, *A. fumigates*, and *F. oxysporum*.	In vitro	[42]
Biological synthesis (Ascorbic acid)	-	Spherical	*A. flavus* and *P. chrysogenum*	In vitro	[79]
Biological synthesis (Green and black teas)	26–40	Spherical	*A.flavus* and *A. parasiticus.*	In vitro	[90]
Biological synthesis (Ajwain and neem)	68	-	*C. musae*	In vitro	[92]
Biological synthesis (Ascorbic acid)	200–500	Faceted	*F. solani*, *Neofusicoccum* sp., and *F. oxysporum.*	In vitro	[152]
Biological synthesis (Ascorbic acid)	200–500	Faceted	*F. oxysporum* f. sp. *Lycopersici*	In vitro and in vivo	[153]
Biological synthesis (*C. paniculatus*)	5	Spherical	*F. oxysporum*	In vitro	[154]
Biological synthesis (*T. pinophilus*)	10	Spherical	*A. niger*, *A terreus*, and *A.fumigatus*	In vitro	[155]
Biological synthesis (*S. capillispiralis*)	3.6–59	Spherical	*Alternariaspp.*, *A. niger*, *Pythium* spp., and *Fusarium* spp.	In vitro	[156]
Biological synthesis (Ascorbic acid)	53–174	Spherical	*F. oxysporum* and *P. capsici*	In vitro	[157]
Chemical synthesis (Chemistry reduction)	20–50	Spherical	*Fusarium* sp.	In vitro	[158]
Chemical synthesis (Chemistry reduction)	-	-	*A. niger*	In vitro	[159]
Chemical synthesis (Hydrothermal)	14 ± 2	Spherical	*A. niger* and *A. oryzae*	In vitro	[160]
Chemical synthesis (Hydrothermal)	30–300	Spherical	*A. alternata*, *A solani*, *F. expansum*, and *Penicilliun* sp.	In vitro	[161]
Chemical synthesis (Chemistry reduction)	3–30	Spherical	*F. equiseti*, *F. oxysporum*, and *F. culmorum*	In vitro	[162]
Chemical synthesis (Chemistry reduction)	25–35	Spherical	*B. cinerea*	In vitro and in vivo	[163]
Chemical synthesis (Chemistry reduction)	14–37	Truncated octahedrons	*F.oxysporum*	In vitro	[164]
Commercial	25	-	*B. cinerea*, *A. alternata*, *M. fructicola*, *C. gloeosporioides*, *F. solani*, *F. oxysporum* f. sp. *Radicis* *Lycopersici*, and *V. dahliae*	In vitro and in vivo	[139]
Commercial	-	-	*R. solani*, *F. oxysporum*, *F. redolens*, *P. cactorum*, *F. hepática*, *G. frondosa*, *M. giganteus*, and *S. crispa*	In vitro	[140]
Commercial	20–30	-	*S. carvi*	In vitro and in vivo	[142]
Commercial	20	Spherical	*A. alternata* and *B. cinerea.*	In vitro	[145]
Commercial	25	-	*B. cinerea*	In vitro and in vivo	[165]

## Data Availability

Not applicable.
